# Regulatory role of short-chain fatty acids in inflammatory bowel disease

**DOI:** 10.1186/s12964-022-00869-5

**Published:** 2022-05-11

**Authors:** Zhilin Zhang, Huan Zhang, Tian Chen, Lin Shi, Daorong Wang, Dong Tang

**Affiliations:** 1 Clinical Medical College, Yangzhou University, Yangzhou, Jiangsu Province People’s Republic of China; 2 Department of General Surgery, Institute of General Surgery, Clinical Medical College, Yangzhou University, Northern Jiangsu People’s Hospital, Yangzhou, 225001 People’s Republic of China

**Keywords:** Inflammatory bowel disease, Immunomodulating activity, Therapeutic effects, Short-chain fatty acids, Gut microbiota

## Abstract

Inflammatory bowel disease (IBD) comprises a group of chronic inflammatory disorders of the gastrointestinal tract. Accumulating evidence shows that the development of IBD is always accompanied by the dysbiosis of the gut microbiota (GM), causing a decrease in prebiotic levels and an increase in harmful metabolite levels. This leads to persistent immune response and inflammation in the intestine, greatly impairing the physiological function of the gastrointestinal tract. Short-chain fatty acids (SCFAs) are produced by probiotic gut bacteria from a fiber-rich diet that cannot be digested directly. SCFAs with significant anti-inflammatory functions regulate immune function and prevent an excessive immune response, thereby delaying the clinical progression of IBD. In this review, we summarize the generation of SCFAs and their potential therapeutic effects on IBD. Furthermore, we suggest that SCFAs may modulate innate immune recognition and cytokine production to intervene in the progression of IBD. Additional randomized controlled trials and prospective cohort studies should also investigate the clinical impact of SCFA.

**Video Abstract**

**Supplementary Information:**

The online version contains supplementary material available at 10.1186/s12964-022-00869-5.

## Background

Inflammatory bowel disease (IBD) is an autoimmune disease with two main clinical forms: Crohn's disease and ulcerative colitis (UC). IBD is currently incurable and affects the quality of life of an increasing number of people [1]. The pathogenesis of IBD is still unclear. It has been suggested that the development of IBD is related to genetics, gut microflora, and dietary habits [2, 3]. The use of agents blocking cytokines, such as tumor necrosis factor (TNF), suppresses the body's immune system and is clinically effective in patients with IBD. However, some cytokines that play a prominent role in the pathogenesis of patients with IBD, such as interleukin (IL)10 and IL17, do not have effective targeting agents [4, 5].

Short-chain fatty acids (SCFAs) produced from dietary fiber in the gut are increasingly favored by researchers for their excellent anti-inflammatory and anticancer effects [6]. Recent studies have shown that SCFAs play an active role in the treatment of inflammation-related diseases, such as hypertension, coronary artery disease, and the development of IBD [7, 8]. SCFAs may be excellent options for the prevention and mitigation of IBD. This article reviews the positive therapeutic effects of SCFAs in IBD and focuses on their modulatory effects on innate immune recognition and cytokine networks.

## Levels of SCFAs in the gut are linked to the development of IBD

**Fig. 1:**
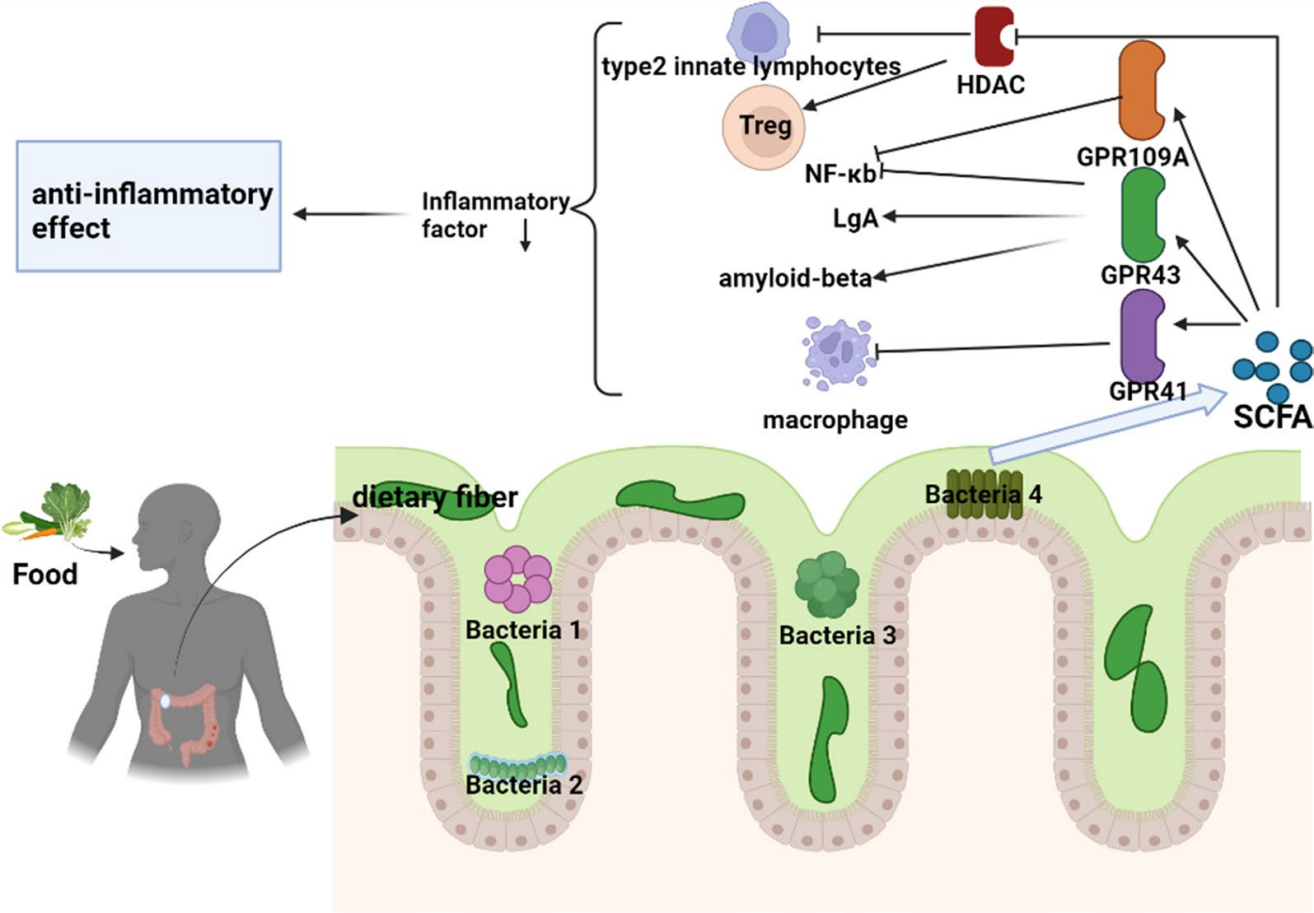
Dietary fiber is digested by intestinal microorganisms to form short-chain fatty acids, which exert anti-inflammatory activities through the G protein-coupled receptor pathway and histone acetylase. The cytokines refer to interleukin 23, interleukin 17 and interleukin beta, etc. They are produced by activation of innate and adaptive immunity after the microbiota is recognized by the immune system and are important contributors to the development of IBD. The Figures in this review were created with BioRender.com

SCFAs, produced by gut microbes that metabolize dietary fiber, exhibit excellent anti-inflammatory effects (Fig. 1)—SCFAs have short chain length (not exceeding 6 carbon atoms) [9]. Prebiotic or microbially accessible carbohydrates, including plant-derived inulin, polysaccharides, and resistant starch, are substrates for SCFA synthesis [10]. However, the three main SCFAs with the highest abundance in the human gut are acetate, propionate, and butyrate. The ratio of their concentrations is 3:1:1 [11, 12]. A high fiber diet can produce approximately 400–800 mmol of SCFAs per day. The concentration and relative proportion of each SCFA in the intestine depends on the microbiota composition, substrate type, and gut transport time [12, 13]. SCFAs are mainly absorbed by colonic epithelial cells and provide energy for their vital activities [14, 15]. A small proportion of SCFAs that are not absorbed by the intestinal epithelium may exert anti-inflammatory, anticancer, and immunomodulatory functions in the gut [16]. Unused SCFAs are excreted in the feces and urine, although these are typically only about 5% of the total SCFAs [17, 18].

The production of SCFAs is mainly regulated by the gut microbiota (GM), with Firmicutes mainly synthesizing butyrate and Bacteroides mainly synthesizing acetate and propionate [12]. Recent studies have shown that there are significant differences in gut microbial species, microbial diversity, and metabolic pathways between patients with IBD and healthy individuals [19, 20]. SCFAs are key prebiotics for maintaining intestinal health and their levels are significantly reduced in the feces of adult patients with IBD. SCFAs are involved in the development and progression of IBD [21]. Analysis of fecal microbiota composition by whole-genome birdshot sequencing previously revealed that the levels of microorganisms associated with SCFA production were substantially reduced in adult patients with IBD [22]. A human study showed that the levels of butyrate producing *Faecalibacterium prausnitzii* were reduced in patients with IBD [23]. Moreover, studies have shown that sodium butyrate supplementation has a positive clinical impact on patients with IBD [24]. In addition, impaired intestinal absorption of SCFAs may precede GM disorders and lead to the development of IBD [25, 26]. Before the loss of butyric acid-producing probiotics, researchers observed impaired oxidation of butyric acid at the intestinal mucosa level, which was also verified at the gene expression level [27, 28].

## Anti-inflammatory mechanism of SCFAs

G protein-coupled receptors (GPCRs) are one of the major pathways that transduce signals from SCFAs, including GPR41, GPR43, and GPR109 [29]. The agonistic activity of SCFAs on GPR41 and GPR43 changes depends on concentration and is correlated with SCFA chain length. GPR43 is more active against acetate and propionate, while GPR41 is more active against SCFAs with long carbon chains [30–32]. GPR41 and GPR43 can almost be activated by all kinds of SCFAs, whereas GPR109a is mainly activated by butyrate and nicotinate [29, 33]. Recent studies have shown that, in addition to the gut, GPCR signaling can improve diseases of multiple body systems, including those of the nervous, cardiovascular, and respiratory systems [34–36]. GPCR signaling plays an anti-inflammatory role in a variety of inflammatory diseases, as shown in Table 1.

**Table 1:** GPCRs signaling inhibits inflammatory diseases

Inflammatory diseases	GPCRs	Functions
Inflammatory bowel disease	GPR43	Promoting the production of IgA, suppressing intestinal inflammation [37, 38]
		Increasing Amphiregulin expression levels in dendritic cells to promote tissue repair [39]
		Inhibiting nuclear factor kappa-B activity [40]
	GPR41	Regulating macrophage activity [41]
	GPR109a	Inhibiting AKT and NF-κB p65 signaling pathways [42]
		Inhibiting IL-23 production [43]
		Improving pathological angiogenesis and inflammatory changes [44]
Diabetic nephropathy	GPR43	Inhibiting high glucose-induced NF-κB activation and oxidative stress [45]
	GPR43 and GPR109A	Inhibiting inflammation in renal tubular cells and podocytes under hyperglycemic conditions [46]
Vascular inflammation	GPR41 and GPR43	Inhibiting pro-inflammatory cytokine production in LPS- or TNFα-stimulated HUVECs [47]
	GPR109A	Playing an anti-atherosclerotic role [48]
Nonalcoholic fatty liver disease	GPR43	Inhibiting hepatic steatosis [37]
Rheumatoid arthritis	GPR43	Significantly inhibiting the expression of key inflammatory factors in rheumatoid arthritis [49]
Osteoarthritis	GPR43	Reducing the expression levels of pro-inflammatory mediators, pro-inflammatory adipokines, and adhesion molecules in chondrocytes [50]
Chronic rhinosinusitis	GPR41 and GPR43	Reducing extent of fibrin deposition and growth of nasal polyps [51]
Alzheimer's disease	GPR41	Inhibiting the ERK/JNK/NF-κB pathway to exert anti-neuroinflammatory effects [52]
	GPR43	Promoting amyloid-beta clearance and inhibiting cellular senescence [53]
	GPR109A	Protecting neurons [54]

In addition, the extremely small size of the SCFAs allows them to enter the nucleus directly and act as inhibitors of histone deacetylase (HDAC) [55]. Histone acetylation is a process that can promote the loosening of the chromatin structure of target genes to enhance gene transcription, but HDAC inhibits this process [56]. SCFAs suppress inflammatory diseases in the body through the HDAC pathway, the inactivation of which leads to the development of autoimmune diseases. SCFAs promote the differentiation of regulatory T cells (Tregs) by inhibiting HDAC activity, and Tregs secrete protective cytokines, such as IL10, to suppress inflammation [57]. Butyrate inhibits the release of inflammatory factors by inhibiting HDAC activity in type 2 innate lymphocytes, ultimately improving airway inflammation [58].

## SCFAs regulate the recognition of innate immune sensors to influence the occurrence of intestinal inflammation

**Fig. 2:**
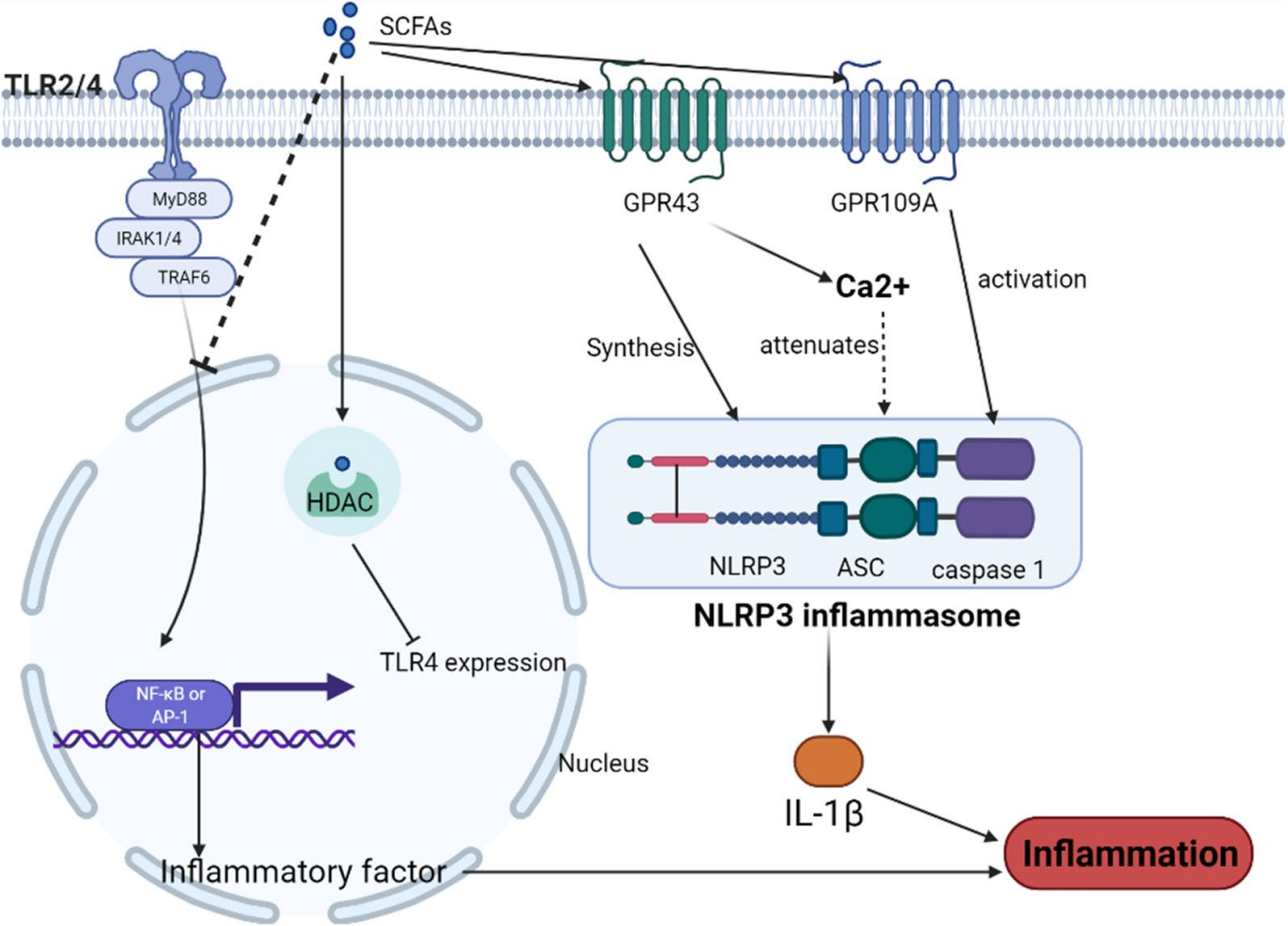
Short-chain fatty acids (SCFAs) inhibit the progression of inflammatory bowel disease (IBD) by regulating innate immune sensors Toll-like receptors (TLRs) and nucleotide-binding and oligomerization domain-like receptor family pyrin domain-containing 3 (NLRP3) inflam-masomes and play a role in inhibiting the progression of IBD. SCFAs not only inhibit TLR sig-naling but also inhibit TLR4 expression by suppressing the histone acetylation pathway. SCFAs also prevent the progression of IBD by regulating the assembly and attenuation of NLRP3 in-flammasomes. The Figures in this review were created with BioRender.com

The crosstalk between innate immunity and microbes is an important cause of altered GM and persistent intestinal inflammation [59]. Intestinal epithelial cells (IECs) are the mainstay of innate immunity in the gut [60]. Recognition of microorganisms by innate immune cells, such as IECs, is the beginning of activation of the innate immune system, and the main recognition of the immune system occurs through pattern recognition receptors (PRRs) [61]. PRRs are important mediators of communication between the immune system and microorganisms, and disturbances in their signaling can lead to dysregulation of the intestinal microbiota and the development of IBD [62, 63]. However, SCFAs can regulate the recognition of the innate immune system, which plays an important role in suppressing intestinal inflammation (Fig. 2).

### Toll-like receptors (TLRs)

TLRs are the typical PRRs associated with IBD development [60]. TLRs are type I transmembrane receptors that are widely expressed in all types of immune cells. After the recognition of microorganisms, TLRs dimerize to activate downstream adapters [64]. The adapter protein transmits the signal into the cell to eventually activate the transcription factors nuclear factor kappa-B and activator protein-1, leading to the expression of inflammatory factors [65]. In addition, TLRs facilitate antigen presentation by DCs and assist in the initiation of adaptive immunity [66]. The activation of these mechanisms leads to inflammation. However, there is a strong correlation between TLR-related inflammation and IBD, and whether TLRs have a protective or destructive effect on the intestinal tract remains controversial [67, 68]. The expression levels of some TLRs in active UC are elevated and accompanied by enhanced signaling, while those are attenuated in the quiescent phase [69]. In contrast, TLR signaling is required for the salvaging of colonic injury in mice [70]. We believe that the complex relationship between TLR signaling and IBD may be due to the following reasons: (1) TLRs are in a highly sensitive state and are extremely susceptible to activation, resulting in the development of persistent inflammation; (2) when TLR signaling is too weak, it tends to lead to an imbalance of the intestinal microbiota and damage of the intestinal mucosa, ultimately leading to inflammation. In conclusion, controlling the TLR signal intensity within a relatively stable range is beneficial to stop the occurrence of IBD.

SCFAs can suppress intestinal inflammation by inhibiting the excessive signaling of TLRs. A high intake of dietary fiber increases the level of SCFAs in the gut and is effective in reducing the extent of TLR-mediated inflammation [71]. TLR4 and TLR2 may be key targets for IBD prevention by SCFAs [72]. Sodium butyrate acts as an HDAC inhibitor to suppress TLR4 expression [73]. In patients with IBD, butyrate inhibits the TLR2-mediated release of inflammatory factors [74, 75]. Butyric acid also reduces adapter protein expression level [76]. In addition, increased level of TLR4 signaling also reduces the abundance of SCFA-producing flora in the gut, which is highly relevant to the development of IBD [75].

### NLRP3 inflammasomes

Nucleotide-binding and oligomerization domain-like receptor family pyrin domain-containing 3 (NLRP3) inflammasome, another innate immune recognition medium, consists of sensor NLRP3, adapter apoptosis-associated spot-like protein, and effector protein cysteinase [77], and it can trigger the activation of caspase-1 to mediate the release of IL1β [78]. This inflammatory response is also closely related to the inflammatory response of the gut. The role of NLRP3 in IBD has been under debate, with early studies suggesting that NLRP3 activation mediates IBD, and more recent studies suggesting that NLRP3 activation inhibits the development of IBD [78]. However, SCFAs regulate the NLRP3 inflammasome and prevent the development of IBD. In a previous study, SCFAs activated the NLRP3 inflammasome by binding to the receptors GPR43 and GPR109A, ultimately maintaining the health of the guts of mice [79]. Further studies have shown that SCFAs maintain intestinal health by regulating NLRP3 inflammasome assembly and attenuation. The activation of GPR43 by SCFAs is required for adequate inflammatory vesicle assembly and IL1β production, which lead to inflammation [80], and GPR43 promotes NLRP3 inflammasome attenuation through a Ca^2+^-dependent mechanism to suppress inflammation [81].

## SCFAs promote intestinal barrier stability by regulating cytokine networks

The intestinal epithelial barrier is the first line of defense of the intestinal immune system and is composed of IECs and the mucus layer [82]. Damage to the intestinal epithelial barrier leads to bacterial translocation and ongoing inflammation and is one of the stages in the development of IBD and colon cancer. The mucus layer is a two-layered structure composed of mucin, with the loose outer layer providing a habitat for intestinal commensal bacteria and the dense inner layer preventing the downward invasion of bacteria [83]. IECs can differentiate into multiple subtypes, including IECs, cupped cells, panniculocytes, and intestinal endocrine cells, of which cupped cells secrete mucin to form the mucus layer [84]. IECs are interconnected by tight junctions (TJs) [85]. Any structural or functional disruption of the intestinal epithelial barrier has the potential to lead to IBD.

### Defenders of the intestinal epithelial barrier: The IL10 family

Cytokines of the IL10 family have broad immunosuppressive effects and may prevent tissue damage caused by excessive inflammatory responses. IL10 family cytokines share similar structures, common receptors, and downstream signaling [86]. Studies have shown that IL10 and IL22 play an important role in maintaining the health of the epithelial barrier of the intestine, and they are often deficient in patients with IBD [87, 88]. There are significant differences in the expression patterns of *IL10* and *IL22* between patients with IBD and healthy individuals [89, 90]. As a key system in the pathogenesis of IBD, the IL10 family has also been verified in animal models. *IL10*-deficient mice exhibit spontaneous IBD [91]. *IL22* gene delivery rapidly ameliorates colonic inflammation in IBD mice [92].

Both IL10 and IL22, when acting on IECs, activate STAT3 signaling in the cells, a key pathway for maintaining intestinal epithelial repair [86, 93]. Studies have shown that SCFAs induce activation of STAT3 signaling in IECs [94]. IL10 and IL22 activate STAT3 in intestinal epithelial stem cells and promote epithelial regeneration [95, 96]. In contrast, STAT3 signaling-deficient mice show a high susceptibility to IBD [97]. In addition, IL10 and IL22 play a beneficial regulatory role on other parts of the intestinal epithelial barrier. Both IL10 and IL22 can upregulate the expression of TJ proteins [98, 99]. Moreover, IL22 also enhances AMP expression and improves the ability of the gut to resist bacterial translocation [100].

However, IL10 and IL22 can both be induced by SCFAs and act to delay the development of IBD. Dietary fiber intake in IL10 knockout mice suppresses colitis [101]. Pentanoate can activate mechanistic target of rapamycin (mTOR) signaling in lymphocytes to promote IL10 production [102]. B lymphocyte-induced maturation protein 1 (Blimp-1) plays a key role in the production of IL10 in Th1 cells. SCFAs activate mTOR and STAT3 signaling in Th1 cells, which activates Blimp-1 protein, leading to IL10 transcription [103–105]. Activation of mTOR and STAT3 signaling appears to play a key role in the regulation of IL10 and IL22 production by SCFAs. SCFAs promote the expression of hypoxia-inducible factor 1α (HIF1α), which ultimately leads to the transcription of IL22, by activating mTOR and STAT3 signaling in CD4 + T cells and ILC3. However, SCFAs enhance the affinity of HIF-1α for the IL22 promoter by inhibiting HDAC [106]. In addition, activation of GPR43 leads to AKT and ERK signaling to promote ILC3 proliferation, which leads to increased expression level of IL22 [107].

### Disruptor of the intestinal epithelial barrier: IL17

IL17A and IL17F, cytokines of the IL17 family, are evolutionarily highly conserved cytokines associated with autoimmune diseases [108]. They are mainly secreted by Th17. Moreover, IL17A and IL17F levels are higher in IBD tissues than in healthy tissues and are accompanied by an increase in the proportion of IL17-producing cells [109, 110]. Inhibition of IL17 expression in a mouse model significantly suppressed colitis [111]. IL17 signaling mediates tissue damage during IBD and has been identified as a highly promising target for IBD intervention [4, 112].

SCFAs engage in regulating the differentiation of CD4 + T cells toward Th17 and Tregs. Th17 expresses high levels of IL17, which promotes inflammation and is directed by the transcription factor retinoic acid-related orphan receptor γt (RORγt) for differentiation [113]. In contrast, Tregs mainly express IL10-like inflammatory factors and are directed to differentiate by the transcription factor forkhead box P3 (Foxp3) [114]. Naive CD4 + T cells express both Foxp3 and RORγt cytokines and the final direction of differentiation depends on the cytokines in the microenvironment [115]. Studies have shown that SCFAs play an important role in the regulation of intestinal T-cell homeostasis; SCFAs promote Treg production and inhibit Th17 production [116]. SCFAs enhance the activity of Foxp3 by inhibiting HDAC [57]. The exon 2 region of Foxp3 interacts directly with RORγt, inhibiting Th17 differentiation and promoting Treg differentiation [117], and this leads to a large reduction in local IL17 concentration in the gut and reduced extent of intestinal inflammation [118].

## Immunonutrition therapy is a viable option for IBD

Based on the remarkable contribution of IL10 in suppressing colonic inflammation in mice, there is a consensus to use recombinant IL10 to treat IBD. However, in a double-blind trial, the rhuIL-10 treatment group showed no significant difference [119]. This may be due to the relatively low bioavailability of IL10 to the intestinal mucosa [120]. To overcome this difficulty, researchers attempted to engineer IL10-producing probiotics to deliver IL10; however, this too has been unsuccessful [88, 121]. Moreover, IL10 may promote the development of cancer to some extent. In conclusion, there is still no effective IL10 treatment available for IBD [122].

Although IL17 plays an important role in the development of IBD, the use of IL17 blockers in the clinical treatment of IBD has not yet yielded effective results and has even exacerbated IBD [4, 112, 123]. IL17A inhibits spontaneous colitis in IL10-/- mice via the inducible nitric oxide synthase pathway. Ablation of IL17A leads to severe colitis [124]. IL17 blockers have been used to treat patients with psoriasis, which can exacerbate IBD [125]. These results suggest that the presence of IL17A may also prevent further deterioration of IBD. The use of IL17A as a target for the treatment of IBD may require a more modest approach.

**Fig. 3:**
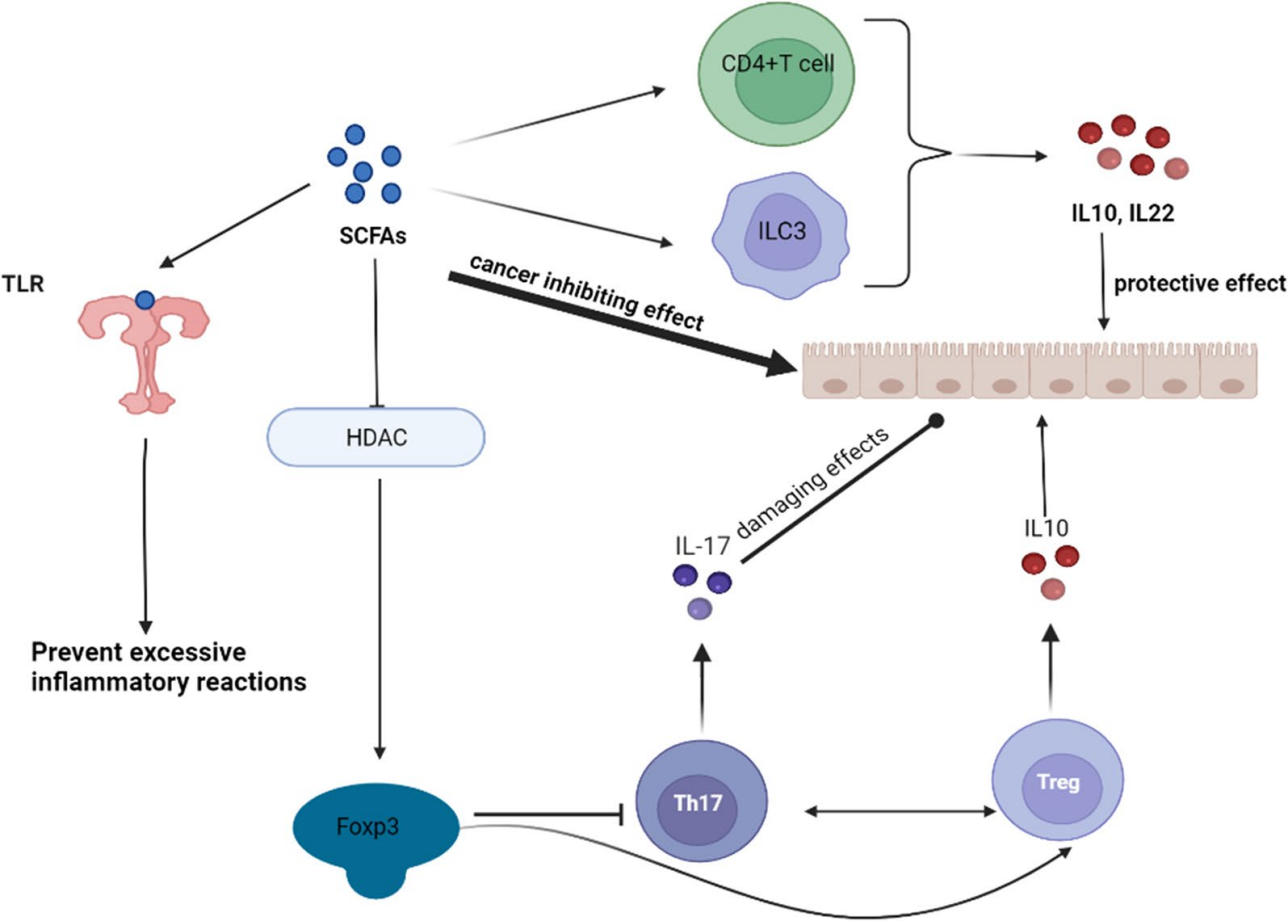
Short-chain fatty acids (SCFAs) are microbial products that can be applied as potential immunonutrition therapies for inflammatory bowel diseases. SCFAs can prevent the development of excessive immune responses by modulating the recognition function of innate immunity, and SCFAs can also play a role in protecting the intestinal barrier by promoting the production of interleukin (IL)10 and inhibiting that of IL17. In addition, SCFAs have excellent anticancer effects. The Figures in this review were created with BioRender.com

SCFAs, the microbial products that are beneficial to the host, can be supplemented through the diet. It not only regulates the recognition of innate immune sensors in the gut, but also the cytokine network in the gut, thus, achieving the goal of stopping the over-reaction of the immune system and inhibiting progression (Fig. 3). The main reasons are as follows: (1) SCFAs can regulate TLRs and NLRP3 inflammasomes to prevent the occurrence of excessive inflammatory responses; (2) SCFAs can promote the expression of IL10 and inhibit the expression of IL17. They can effectively prevent cancer due to repeated damage-repair on top of the treatment of IBD; (3) the ability of SCFAs to promote apoptosis and inhibit the activity of cancer cells has been reviewed in many studies [126, 127]; (4) in addition, no side effects have been observed with SCFAs for the treatment of IBD.

## Outlook: SCFAs have a potential therapeutic effect on IBD

As an autoimmune disease, IBD has a serious impact on the quality of life of patients, but there is no cure for this disease. The pathogenesis of IBD is still unclear and it is now understood that the development of IBD is associated with genetic factors, dietary habits, and intestinal flora. Targeted inflammatory cytokine blockers, such as TNF and JAK blockers, have been clinically effective in relieving symptoms in patients with IBD. However, they also increase the probability of patients developing infectious diseases [128]. Some cytokines, such as IL10 and IL17, play a prominent role in the development of IBD, yet there are no effective IL10/17-targeted drugs available for clinical use. SCFAs, which are the microbial products that are beneficial to the host, can be supplemented through the diet. This not only regulates the recognition of innate immune sensors in the gut but also the cytokine network in the gut, thus, achieving the goal of stopping the over-reaction of the immune system and inhibiting the progression of IBD. Moreover, SCFAs also play anticancer roles and can effectively stop the development of cancer in patients with IBD. In a preliminary double-blind, placebo-controlled study, Facchin et al. demonstrated that sodium butyrate supplementation increased the growth of SCFA-producing bacteria and improved the inflammatory response in patients with IBD [24]. However, other randomized controlled trials and prospective cohort studies should also investigate the clinical impact of SCFA as one of the future directions to improve the quality of life of patients with IBD.

## Data Availability

Not applicable.

## Abbreviations

AMP: Antimicrobial peptides
AP-1: Activator protein 1
Blimp-1: B lymphocyte-induced maturation protein 1
Foxp3: Forkhead box P3
GM: Gut microbiota
GPCRs: G protein-coupled receptors
HDAC: Histone deacetylase
HIF1α: Hypoxia-inducible factor 1α
IBD: Inflammatory bowel disease
IEC: Intestinal epithelial cells
IL10: Interleukin 10
iNOS: Inducible nitric oxide synthase
mTOR: Mechanistic target of rapamycin
NF-κB: Nuclear-factor kappa-B
NLRP3: Nucleotide-binding and oligomerisation domain-like receptor familiy pyrin domain- containing3
PRR: Pattern recognition receptors
RORγt: Retinoic acid-related orphan receptor γt
SCFA: Short chain fatty acids
TJ: Tight junctions
TNF: Tumor Necrosis Factor
TLRs: Toll-like receptors
Treg: Regulatory T cells
UC: Ulcerative colitis
